# Morbidity and mortality of nonagenarians undergoing CoreValve implantation

**DOI:** 10.1186/1471-2261-12-80

**Published:** 2012-09-24

**Authors:** Ibrahim Akin, Stephan Kische, Lylia Paranskaya, Henrik Schneider, Tim C Rehders, Gökmen R Turan, Dimitar Divchev, Gunther Kundt, Ilkay Bozdag-Turan, Jasmin Ortak, Ralf Birkemeyer, Christoph A Nienaber, Hüseyin Ince

**Affiliations:** 1 Heart Center Rostock, Department of Internal Medicine I, University Hospital Rostock, Rostock School of Medicine, Ernst-Heydemann-Str. 6, 18057, Rostock, Germany; 2 Institute for Biostatistics and Information in Medicine and Ageing Research, Rostock School of Medicine, University Hospital Rostock, Ernst-Heydemann-Str 6, 18057, Rostock, Germany

**Keywords:** CoreValve, Aortic stenosis, Nonagenarian, Surgery, TAVI

## Abstract

**Background:**

Nonagenarians are mostly denied from different therapeutic strategies due to high comorbidity index and risk-benefit calculation. We present the results of nonagenarians with high comorbidity index not eligible for conventional aortic valve surgery undergoing transcatheter aortic valve implantation (TAVI) with the CoreValve system.

**Methods:**

Our retrospective analysis include baseline parameters, procedural characteristics, morbidity, mortality as well as twelve-lead surface ECG and echocardiographic parameters which were revealed preinterventionally, at hospital discharge and at 30-day follow-up. Clinical follow-up was performed 6 months after TAVI.

**Results:**

Out of 158 patients 11 nonagenarians with a mean age of 92.6 ± 1.3 years suffering from severe aortic valve stenosis and elevated comorbidity index (logistic EuroSCORE of 32.0 ± 9.5%, STS score 25.3 ± 9.7%) underwent TAVI between January 2008 and January 2011 using the third-generation percutaneous self-expanding CoreValve prosthesis. Baseline transthoracic echocardiography reported a mean aortic valve area (AVA) of 0.6 ± 0.2 cm^2^ with a mean and peak pressure gradient of 60.2 ± 13.1mmHg and 91.0 ± 27.4mmHg, respectively. The 30-day follow up all cause and cardiovascular mortality was 27.3% and 9.1%, respectively. One major stroke (9.1%), 2 pulmonary embolisms (18.2%), 1 periprocedural (9.1%) and 1 (9.1%) spontaneous myocardial infarction occured. Life-threatening or disabling bleeding occurred in 2 cases (18.2%), and minor bleeding in 7 cases (63.6%). Mean severity of heart failure according to NYHA functional class improved from 3.2 ± 0.8 to 1.36 ± 0.5 while mean AVA increased from 0.6 ± 0.2cm^2^ to 1.8 ± 0.2cm^2^. At 6-months follow-up 8 patients (72.7%) were alive without any additional myocardial infarction, pulmonary embolism, bleeding, or stroke as compared to 30-day follow-up.

**Conclusion:**

Our case series demonstrate that even with elevated comorbidity index, clinical endpoints and valve-associated results are relatively favorable in nonagenarians treated with CoreValve.

## Background

Aortic valve stenosis (AS) is the most common native valve disease, becoming more prevalent with the ageing population [1]. The only definitive therapy for patients with severe symptomatic AS consists of surgical valve replacement [1]. However, conventional valve surgery is associated with high mortality and morbidity for some patients due to high comorbidity index. This leads to conservative management of these patients despite the poor prognosis associated with medical therapy [2]. Since its introduction in 2002 [3], TAVI proves to be a robust technique in the treatment of severe symptomatic AS in high surgical risk patients [4-7]. This study firstly evaluated the outcomes of TAVI in nonagenarians presenting a comorbidity index too high for conventional surgical valve replacement.

## Methods

### Patients

In our retrospective analysis, between January 2008 and January 2011, 11 nonagenarians out of a total amount of 158 patients who underwent TAVI using the third-generation percutaneous self-expanding CoreValve prosthesis (Medtronic, Minneapolis, MN, USA) were identified for this case series. The criteria for inclusion and exclusion to the TAVI procedure have been described elsewhere [4-7]. In brief, patients were included with echocardiographic measurements demonstrating severe native valvular AS with an area < 1cm^2^, or < 0.6cm^2^/m^2^ regardless of adjunct regurgitation; a diameter of the basal orifice of the stenosed valve between 20mm and 27mm; and a diameter at the sinutubular junction ≤ 43mm. Most importantly, all patients were considered unfit for open surgery with an EuroSCORE ≥ 20% [4,8]. TAVI was suggested in agreement between a cardiac surgeon and both, a clinical and interventional cardiologist; patient’s or referring physician’s preference was not relevant. Treatment strategy was in compliance with the Helsinki Declaration. Our local ethics committee approved this treatment strategy. Patients gave informed consent prior TAVI. Pacemaker implantation at follow-up was considered indicated in case of complete AV block and type II second-degree AV block.

### Procedure

Details of the implantation procedures have been described elsewhere [4-7]. In brief, all patients were operated in a hybride interventional suite under general anaesthesia to assure stable hemodynamics and minimize patient movement during valve implantation. TAVI was performed via femoral access under fluoroscopic imaging by using the ProStar XL system (Abbott Vascular, Illinois, USA). The aortic valve was initially dilated using a standard valvuloplasty balloon (18-25mm x 40-100mm) with a nominal diameter slightly (2-6mm) smaller than the aortic valve and followed by CoreValve insertion [4-7] and in case of residual aortic insufficiency a post-TAVI balloon dilatation to ensure complete apposition. After procedure patients were transferred to intensive care unit (ICU). To assess conduction disorders, ECG monitoring was continuously performed over 7 days. All patients were prophylactically given a temporary pacemaker via femoral venous access; with VVI mode the active pacing was 40bpm.

### Definitions

Definitions of all clinical outcomes were performed according to the Valve Academic Research Consortium [9].

### Statisitical methods

All data were processed using the SPSS statistical package for windows, release 16.0 (Chicago, Illinois, USA). The descriptive statistical characteristics for quantitative parameters are listed for normally distributed data in mean ± standard deviation and in non-normally distributed data in median with interquartile range.

## Results

Out of 158 patients a total of 11 nonagenarians with a mean age of 92.6 ± 1.3 years underwent TAVI at our institution from January 2008 to January 2011. Clinical presentation was dominated by dyspnoe (100%), angina (36.4%), syncope (36.4%) and acute heart failure (45.5%). The mean New York Heart Association (NYHA) functional class was 3.2 ± 0.8. Many patients suffered from high comorbidity index resulting in a mean logistic EuroSCORE of 32.0 ± 9.5% and a STS score of 25.3 ± 9.7% (Table 1). Baseline transthoracic echocardiography reported a mean AVA of 0.6 ± 0.2 cm^2^ with a mean and peak pressure gradient of 60.2 ± 13.1mmHg and 91.0 ± 27.4mmHg, respectively. Out of 11 patients 9 (81.8%) had mild and 1 (9.1%) had moderate aortic regurgitation, respectively. The analysis of ECGs revealed sinus rhythm in 8 patients (72.7%) and atrial fibrillation in 1 individual (9.1%) (Table 2).

**Table 1:** Baseline characteristics of the study population (n = 11)

**Variable**	
**Clinical parameters**	
Male, n (%)	4 (36.4)
Age (yrs)	92.6 ± 1.3
BMI (kg/m^2^)	28.6 ± 3.0
Hypertension, n (%)	9 (81.8)
Dyslipidemia (%)	9 (81.8)
Smoker, n (%)	3 (27.3)
Diabetes mellitus, n (%)	7 (63.6)
Renal insufficiency (creatinine level > 1.5mg/dl), n (%)	6 (54.5)
Chronic hemodyalisis, n (%)	2 (18.2)
Chronic obstructive pulmonary disease, n (%)	2 (18.2)
New York Heart Association functional class (grade)	3.2 ± 0.8
Logistic EuroSCORE (%)	32.0 ± 9.5
STS score (%)	25.3 ± 9.7
Dyspnoe, n (%)	11 (100)
Angina, n (%)	4 (36.4)
Syncope, n (%)	4 (36.4)
Cardiac decompensation, n (%)	5 (45.5)
Pulmonal artery systolic pressure (mmHg)	40.8 ± 18.7
Porcelain aorta, n (%)	0 (0)
Ischemic heart disease, n (%)	8 (72.7)
Previous percutaneous coronary intervention, n (%)	6 (54.5)
Previous coronary artery bypass graft surgery, n (%)	4 (36.4)
Peripheral vessel disease, n (%)	2 (18.2)
Previous stroke, n (%)	2 (18.2)
**Laboratory findings**	
Hemoglobin (mg/dl)	7.8 ± 1.2
Hematokrit (%)	37.7 ± 4.8
Creatinine (μmol/l)	183.9 ± 223.6
MDRD	48.2 ± 24.6
**Medication**	
Aspirin	7 (63.6)
ACE-Inhibitor	7 (63.6)
ARB	2 (18.2)
Beta-Blocking agent	8 (72.7)
Ca-chanel blocker	3 (27.3)
Antiarythmic agent	0 (0)
Statin	7 (63.6)

**Table 2:** Echocardiographic and electrocardiographic parameters

**Echocardiographic parameters**	
Left ventricular ejection fraction (%)	44.6 ± 7.8
Aortic valve area (cm^2^)	0.6 ± 0.2
Peak pressure gradient (mmHg)	91.0 ± 27.4
Mean pressure gradient (mmHg)	60.2 ± 13.1
Aortic annulus dimension (mm)	20.7 ± 3.5
Aortic bulbus dimension (mm)	29.7 ± 4.1
Left ventriculae end-diastolic diemater (mm)	54.6 ± 7.7
Interventricular septal dimension (mm)	13.2 ± 1.9
Aortic regurgitation, n (%)	10
Grade I	9 (81.8)
Grade II	1 (9.1)
Grade III	0
Mitral insufficiency, n (%)	
Grade I	6 (54.5)
Grade II	4 (36.4)
Grade III	0
**Electrocardiographic parameters**	
Sinus rhythm, n (%)	8 (72.7)
Atrial fibrillation, n (%)	1 (9.1)
Pacemaker, n (%)	2 (18.2)
Heart rate (bpm)	70.1 ± 10.7
PQ interval (ms)	199.4 ± 62.8
QRS width (ms)	112.6 ± 23.4
QT interval (ms)	397.5 ± 30.7
Left bundle branch block, n (%)	0 (0)
Right bundle branch block, n (%)	0 (0)
Left anterior hemiblock, n (%)	2 (18.2)
Left posterior hemiblock, n (%)	0 (0)

TAVI was successfully performed in all patients (100%) and no conversion to surgical AVR was necessary. In 2 cases we had an embolization of the valve. In both cases the valves were not completely released and thus were re-captured and implanted in a proper position. Mean procedure and fluoroscopy time was 110.3 ± 29.5min and 16.0 ± 6.8min, respectively. Mean ICU stay was 3.36 ± 1.9 days, whereas mean hospital stay was 18.5 ± 5.4 days (Table 3).

**Table 3:** Intraoperative data of patients

**Parameter**	
Procedural success, n (%)	11 (100)
Conversion to surgical AVR, n (%)	0 (0)
Intraprocedural circulatory depression, n (%)	1 (9.1)
Catecholamine therapy, n (%)	1 (9.1)
Resuscitation, n (%)	1 (9.1)
Defibrillation, n (%)	1 (9.1)
Vascular access site complication requiring surgery, n (%)	1 (9.1)
Contrast agent (ml)	120.9 ± 44.7
Procedure time (min)	110.3 ± 29.5
Fluoroscopy time (min)	16.0 ± 6.8
CoreValve size (mm)	
26	9 (81.8)
29	2 (18.2)
Pre TAVI valvuloplasty, n (%)	11 (100)
Post TAVI valvuloplasty, n (%)	5 (45.5)
Number of inflations after TAVI (n)	1.2 ± 0.6
Balloon diameter (mm)	20.6 ± 1.8
Balloon length (mm)	62.7 ± 16.8
Valve embolization, n (%)	2 (18.2)
Post TAVI aortic valve mean gradient	5.3 ± 4.1
Angiographic aortic insufficiency (grade)	0.9 ± 0.45
ICU stay (days)	3.36 ± 1.9
Hospital stay (days)	18.5 ± 5.4

All cause and cardiovascular mortality was 18.2% (n = 2) and 9.1% (n = 1), respectively. One major stroke (9.1%), 1 minor stroke (9.1%) and 2 TIAs (18.2%) occurred. One patient each (9.1%) experienced a periprocedural (<72h) myocardial infarction and a spontaneous myocardial infarction, whereas 1 patient (9.1%) was in need for new hemodialysis. Life threatening or disabling bleeding occurred in 2 cases (18.2%), and minor bleeding in 7 cases (63.6%). Three patients were in need for pacing (27.3%) (Table 4).

**Table 4:** In-hospital outcome of patients

**In-hospital outcome**	
Mortality, n (%)	
All cause n (%)	2 (18.2)
Cardiovascular, n(%)	1 (9.1)
Stroke, n (%)	
Major	1 (9.1)
Minor	1 (9.1)
TIA, n (%)	2 (18.2)
Myocardial infarction, n (%)	
Periprocedural (<72h)	1 (9.1)
Spontaneous (>72h)	1 (9.1)
Pneumonia, n (%)	1 (9.1)
Sepsis, n (%)	1 (9.1)
Pulmonary embolism	1 (9.1)
Acute kidney injury, n (%)	
Grade I	3 (27.3)
Grade II	0 (0)
Grade III	1 (9.1)
New need for hemodialysis	1 (9.1)
Bleeding, n (%)	
Life-threatening or disabling	2 (18.2)
Major	0 (0)
Minor	7 (63.6)
Vascular complication	
Major	1 (9.1)
Minor	3 (27.3)
Need for Pacing, n (%)	3 (27.3)
New left bundle branch block	6 (54.6)
New second or third-degree AV block	3 (27.3)
Need for Re-do (TAVI or Surgery)	0 (0)

30-day follow up all cause and cardiovascular mortality was 27.3% (n = 3) and 9.1% (n =1), respectively. One major stroke (9.1%), 2 minor strokes (18.2%) and 3 TIAs (27.3%) occurred. There were 1 periprocedural (<72h) myocardial infarction (9.1%), and 1 spontaneous myocardial infarctions (9.1%) requiring percutaneous coronary intervention (PCI) at a mean follow-up of 13 days. Ten patients (90.9%) presented combined safety endpoints. Mean severity of heart failure according to the NYHA functional class was 1.36 ± 0.5. Transthoracic echocardiography revealed a mean AVA and aortic mean pressure gradient of 1.8 ± 0.2 cm^2^ and 8.3 ± 3.9 mmHg, respectively (Table 5).

**Table 5:** 30-day follow-up of patients

**30-day outcome**	
Mortality, n (%)	
All cause n (%)	3 (27.3)
Cardiovascular, n(%)	1 (9.1)
Stroke, n (%)	
Major	1 (9.1)
Minor	2 (18.2)
TIA, n (%)	3 (27.2)
Myocardial infarction, n (%)	
Periprocedural (<72h)	1 (9.1)
Spontaneous (>72h)	1 (9.1)
New need for hemodialysis	2 (18.2)
Bleeding, n (%)	
Life-threatening or disabling	2 (18.2)
Major	0 (0)
Minor	7 (63.6)
Vascular complication	
Major	1 (9.1)
Minor	3 (27.3)
Pulmonary embolism	2 (18.2)
Combined safety endpoints	10 (90.9)
Need for Pacing, n (%)	5 (45.5)
New left bundle branch block	7 (63.7)
New second or third-degree AV block	5 (45.5)
NYHA functional class	1.36 ± 0.5
Cardiac decompensation, n (%)	2 (18.2)
Syncope, n (%)	0 (0)
Need for hospitalization due to cardiac causes, n (%)	3 (27.2)
Need for Re-do (TAVI or Surgery)	0 (0)
**Echocardiographic parameters**	
Left ventricular ejection fraction (%)	45.6 ± 6.9
Left ventriclura end-diastolic diameter (mm)	50.1 ± 7.1
Aortic valve area (cm^2^)	1.8 ± 0.2
Aortic peak pressure gradient (mmHg)	16.6 ± 6.7
Aortic mean pressure gradient (mmHg)	8.3 ± 3.9
Interventricular septal dimension (mm)	13.5 ± 1.6
Aortic regurgitation, n (%)	
Grade I	9 (81.9)
Grade II	2 (18.2)
Grade III	0 (0)
Pulmonal artery systolic pressure	45.7 ± 14.6
Mitral insufficiency (%)	
Grade I	8 (72.8)
Grade II	2 (18.2)
Grade III	0 (0)

At 6 months follow-up 8 patients (72.7%) were alive without any additional myocardial infarction, pulmonary embolism, bleeding, TIA or stroke. Mean severity of NYHA functional class was 1.34 ± 0.7.

## Discussion

With the ageing population the demand for cardiac operations in elderly patients has steadily increased over the last 10 to 15 years. Moderate-to-severe AS occurs in 5% of individuals 75 to 86 years of age, and critical AS is seen in >5% of those >85 years of age [10]. In-hospital death and stroke rates may be as high as 8.5% and 8%, respectively [11]. Mean duration of postoperative hospital stay in most reports is >2 weeks for very elderly patients, with most being discharged to nursing care facilitates.

Today, about one third of patients with severe AS are not referred for valve replacement surgery because of the risk perceived by both patients and physicians. TAVI is a robust technique, which offers advantages to high surgical risk patients [5-7]. Elderly with AS often present comorbidities, which are responsible for the considerable heterogeneity of operative risk and which hamper the decision, if the beneficial outcome of surgery, compared with spontaneous outcome, outweighs the risk of intervention. However there are no explicit age related restrictions for surgical aortic valve replacement according to guidelines on treatment of severe symptomatic AS [1,11]. In nonagenarians, age alone accounts for a predicted logistic EuroSCORE mortality risk of 6.55% for male patients, as for females it raises to 8.89% without any other preoperative risk factors [8]. In our series predicted logistic EuroSCORE mortality rate was 32.0 ± 9.5% for whole cohort. Feasibility of TAVI on nonagenarians was proven by the high procedural success rate (100%). The reduction in afterload after TAVI resulted in immediate marked hemodynamic improvement and translated into symptomatic relief, with a reduction in NYHA class by a mean of 1.8 grades. We also show markedly improvements in the AVA from 0.6 to 1.8 cm^2^ and reductions in mean pressure gradients from 60.2 to 8.3 mmHg, which compare favorably to the results for younger patients treated with TAVI [5,6,12,13].

Safety was assessed using primary and secondary endpoints following recommendations of the Valve Academic Research Consortium [9]. As there is no trial exclusively in nonagenarians or subgroup analyses of large trials our outcomes are not comparable with the literature.

All-cause mortality is considered as the “gold standard” in surgical clinical trials with cardiovascular mortality as an important secondary endpoint [4,9,14]. In our study the 30-day follow up all cause and cardiovascular mortality was 27.3% and 9.1%, respectively, which is slightly higher or comparable to 11.4% to 29% all cause mortality for nonagenarians with low comorbidity index as stated by the estimated logistic EuroSCORE treated with surgical aortic valve replacement [15-18] and 5% to 20% all-cause and 10% cardiovascular mortality for TAVI performed at younger patients [5-7].

Like mortality, operative morbidity in the elderly is higher than in younger patients. This is true in particular for the frequency of stroke. Some authors have shown that older age is strongly related to a neurological event, the latter being associated with a previous history of stroke and advanced atherosclerotic disease [19]. We report one major stroke (9.1%) in our cohort, which compares to 2.1% to 8.9% in a nonagenarian population treated with surgical aortic valve replacement [5-7] and 0% to 10% for TAVI performed at younger patients [5-7,13]. Our periprocedural (<72h) (9.1%), and spontaneous (9.1%) myocardial infarction rate is markedly higher compared to 0% to 0.3% for TAVI performed at younger patients [5-7,13] and 5.7% for surgical valve replacement performed at nonagenarians [16] pointing out the high cardiovascular risk profile of our severely ill patients. Two life-threatening or disabling bleeding occurred (18.2%) which compares favorably to rates of 3.6% to 24% for TAVI performed at younger patients [5-7,13]. Vascular complications after TAVI occur with an incidence of 7.5% to 16.3% and remain a significant cause of mortality and morbidity [5-7,13]. We report similar rates of major and minor vascular complications. 3 patients presented acute kidney injury grade I (27.3%), and one patient grad III (9.1%), which is comparable to 6% to 28% of acute kidney injuries reported in the literature [20].

As shown in previous studies, the very elderly are more likely to experience postoperative complications, and consequently, a prolonged hospitalization and intensive therapy [21,22]. However, the length of hospital and ICU stay reported in our study (18.5 ± 5.4 and 3.36 ± 1.9 days respectively) is comparable to data for younger patients (7 to 17 days and 2.8 days respectively [23].

In general population, life expectancy in nonagenarians is 2.5 and 3.5 years for men and women, respectively [24]. Although one should take into account the very advanced age and frailty of these subjects in the interpretation of such results, all-cause death is still not low despite successful TAVI. However, the patients who survived the follow-up achieved in most cases an improvement in their clinical symptoms. Thus, TAVI in the very elderly have the potential to significantly ameliorate functional status and quality of life, at the price of considerable periinterventional and follow-up mortality rates. The risk-benefit balance of TAVI of nonagenarians should then be considered to be paradigmatically different than in the younger candidates, in whom the prolongation of life is usually considered the primary aim. The clinician should consider these findings while evaluating the very elderly for TAVI and properly discuss these issues in the light of the patient’s expectations.

## Conclusions

Our case series demonstrate that even with elevated comorbidity index clinical endpoints and valve-associated results are relatively favorable in nonagenarians treated with CoreValve prosthesis. Due to the small sample size there is a need for more data from multicenter large-scale trials or registries to elucidate the role of TAVI in nonagenarian patients.

## Abbreviations

AS: Aortic stenosis; AVA: Aortic valve area; AVR: Aortic valve replacement; ECG: Electrocardiography; ICU: Intensive care unit; NYHA: New York Heart Association; PCI: Percutaneous coronary intervention; TAVI: Transcatheter aortic valve implantation.

## Competing interests

The authors declare that they have no competing interests.

## Authors' contributions

IA, SK, HI, HS, TCR, LP, DD, GRT, IBT, JO, RB, CAN participate in treating the patients during intervention/ICU and acquisition of data. IA, HI and CAN wrote the manuscript. IA and GK performed the statistical analyses. All authors read and approved the final manuscript.

## Pre-publication history

The pre-publication history for this paper can be accessed here:

http://www.biomedcentral.com/1471-2261/12/80/prepub
